# Caudal lumbar subarachnoid diverticulum in a Cockapoo

**DOI:** 10.1093/jvimsj/aalag014

**Published:** 2026-02-14

**Authors:** Joe Poacher, Paul Freeman

**Affiliations:** The Queen’s Veterinary School Hospital, University of Cambridge, Department of Clinical Veterinary Medicine, Cambridge CB30ES, United Kingdom; The Queen’s Veterinary School Hospital, University of Cambridge, Department of Clinical Veterinary Medicine, Cambridge CB30ES, United Kingdom

**Keywords:** subarachnoid diverticulum, lumbar, intervertebral disc extrusion, dog

## Abstract

We describe a Cockapoo with a subarachnoid diverticulum (Type III), at the level of L6-7. Magnetic resonance imaging identified a circumferential dilatation of the dural sac, extending from the cranial endplate of L6 to the midbody of L7, containing T2-weighted hyperintense and T1-weighted hypointense material that suppressed on fluid-attenuated inversion recovery sequences, consistent with cerebrospinal fluid. An exploratory dorsal laminectomy confirmed a subarachnoid diverticulum (Type III), and a durotomy was performed. After surgical decompression, full clinical resolution was observed. This case had a clinical presentation that mimicked an intervertebral disc extrusion.

## Introduction

A spinal subarachnoid diverticulum (SAD), also referred to as arachnoid diverticulum is a fluid-filled dilatation that arises from the subarachnoid space.^1^ A classification system adapted from human medicine categorizes lesions into 3 types: Type I is extradural without involvement of the nerve roots, Type II is extradural with nerve root involvement, and Type III is intradural.^2^ Despite their subarachnoid origin, Type I and II arachnoid diverticula are extradural in location, and it has been hypothesized that a small dural tear allows cerebrospinal fluid (CSF) pressure to drive herniation of the arachnoid membrane through the defect, forming an extradural pouch.^3^ Pugs, rottweilers, and French bulldogs are overrepresented among dogs with SAD.^1^ Cervical and thoracolumbar localization is most common, and hypothesized to be a consequence of high mobility in these areas.^4–6^ Males also seem to be overrepresented.^4^^,^^6–8^ Historically, it has been thought that larger breed dogs are more prone to cervical lesions and small breeds to thoracolumbar lesions.^5^ Caudal lumbar and lumbosacral localizations are extremely rare,^8^ with a localization of L6-7 only being reported in a single French bulldog.^9^

## Clinical case report

A 4-year-old neutered male Cockapoo (Cocker spaniel-poodle cross) presented with a 1-month progressive history of abnormal gait in the hindlimbs. The onset was insidious, initially manifesting as left hindlimb lameness. The dog was reluctant to jump, climb stairs, and would occasionally toe touch. The owners felt that, as it progressed, the signs occasionally alternated between the left and right hindlimbs. They also noted progression in signs suggestive of pain, including increased lethargy, vocalization when the left leg was touched and shaking the leg.

At the referring veterinary practice, orthopedic examination, including under sedation, was normal. Radiographs of each joint in both hindlimbs were taken and were later reviewed by the orthopedic team at the Queen’s Veterinary School Hospital. They showed very mild hip dysplasia not consistent with the severity of the clinical signs. The dog initially was started on meloxicam (0.1 mg/kg PO q24h). However, because of a lack of clinical response, treatment was changed to prednisolone (1 mg/kg PO q12h) two days before presentation. Exercise also was restricted to 10-minute walks on a lead, and the dog slowed down and appeared more painful toward the end of these walks.

The dog weighed 14.7 kg with a body condition score of 3.5/5. General clinical examination was normal, other than evidence of otitis externa in the right ear, for which the dog was receiving topical treatment (miconazole nitrate, polymyxin B sulfate, prednisolone acetate).

Neurologic examination disclosed normal mentation, persistent 4/10 left hind limb lameness, graded using a 0-10 numerical rating scale (0 = no lameness, 10 = non-weight-bearing), with intermittent limb carrying and signs consistent with monoparesis. When standing, the dog consistently offloaded the left hindlimb. Cranial nerve examination was unremarkable. Postural reactions were normal in all four limbs on paw replacement testing. Withdrawal reflexes were normal in the forelimbs and right hindlimb but decreased in the left hindlimb. The patellar reflex was normal bilaterally. The cutaneous trunci reflex was present but consistently weaker in the caudal lumbar region. Pain was elicited on palpation of the caudal lumbar spine and during left hip extension. Neuroanatomical localization was consistent with a lesion affecting the L4-S3 spinal cord segments or, more likely, spinal nerve roots. The most likely differential diagnosis was considered to be an intervertebral disc extrusion (Hansen Type 1). Other differential diagnoses included inflammatory conditions (eg, discospondylitis, meningoencephalomyelitis of unknown origin) and neoplasia.

**Figure 1:**
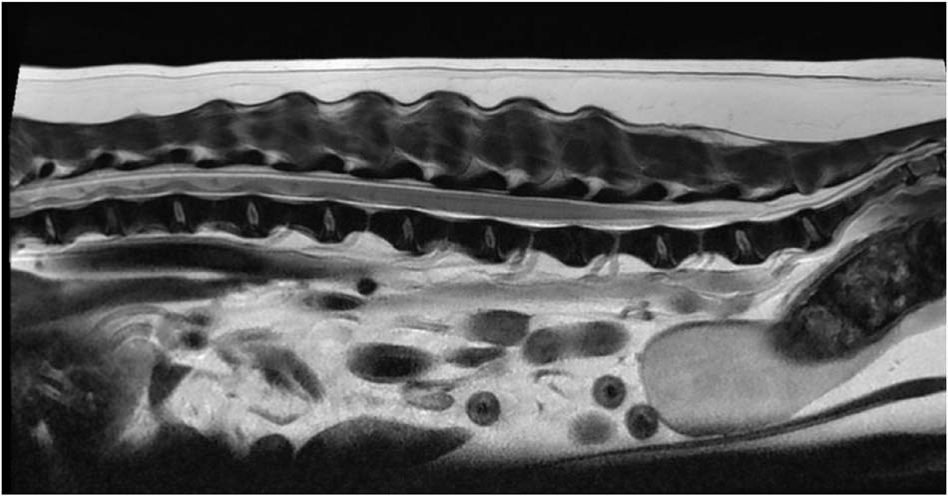
Sagittal T2-weighted image of the spinal cord from T10-S4. Extending from the cranial endplate of L6 to the mid-body of L7, a circumferential dilation of the dural sac can be seen. The dural sac was filled with a T2W hyperintense fluid, consistent with CSF.

**Figure 2:**
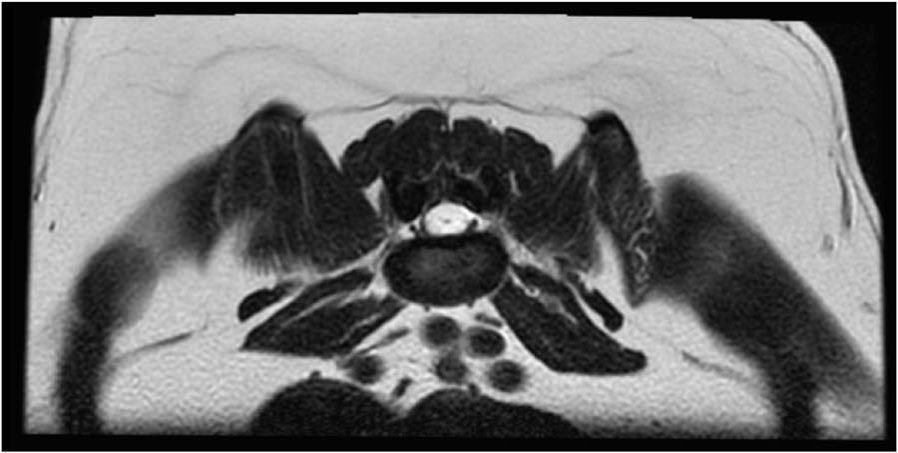
Transverse T2-weighted image of the spinal cord at the level of L6-7 intervertebral disc space. The filium terminale can be seen decreased to a small thread-like structure surrounded by a T2W hyperintense fluid consistent with CSF, later confirmed to be a subarachnoid diverticulum.

Magnetic resonance imaging (MRI) of the L4-S3 spinal cord segments was performed under general anesthesia. At the level of L7, a transitional vertebra was identified, featuring an elongated, partially sacralized transverse process on the left side. This abnormality resulted in slight medial deviation of the left L7 nerve root. However, evidence of thickening or contrast enhancement of the associated nerve root was not observed. At the L7-S1 intervertebral disc space, mild dorsal protrusion of the intervertebral disc was noted, extending into the ventral extradural space. Extending from the cranial endplate of L6 to the mid-body of L7, a circumferential dilatation of the dural sac was noted. The dural sac appeared T1-weighted hypointense and T2W hyperintense and suppressed on fluid-attenuated inversion recovery sequences, consistent with CSF (Figure 1). Mild contrast enhancement of the meninges was observed on subtraction T1W images. At the level of the L6-7 intervertebral disc space, the filum terminale was decreased to a small fine central thread-like structure (Figure 2). At the level of L4, the dorsal aspect of the spinal cord showed irregular margins, with possible fine tethering of the pia mater to the arachnoid or the dura mater. Based on these findings, a SAD (Type III) or caudal lumbar spinal cyst were considered the most likely differential diagnoses.

**Figure 3:**
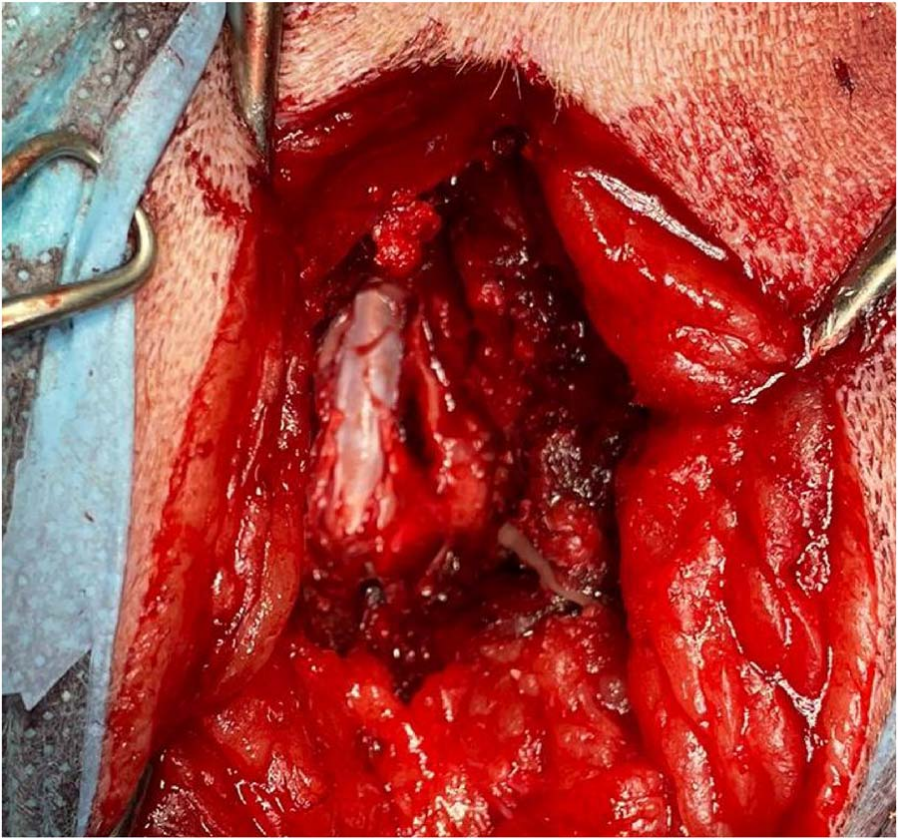
Intraoperative appearance of the subarachnoid diverticulum, after dorsal laminectomy.

**Figure 4:**
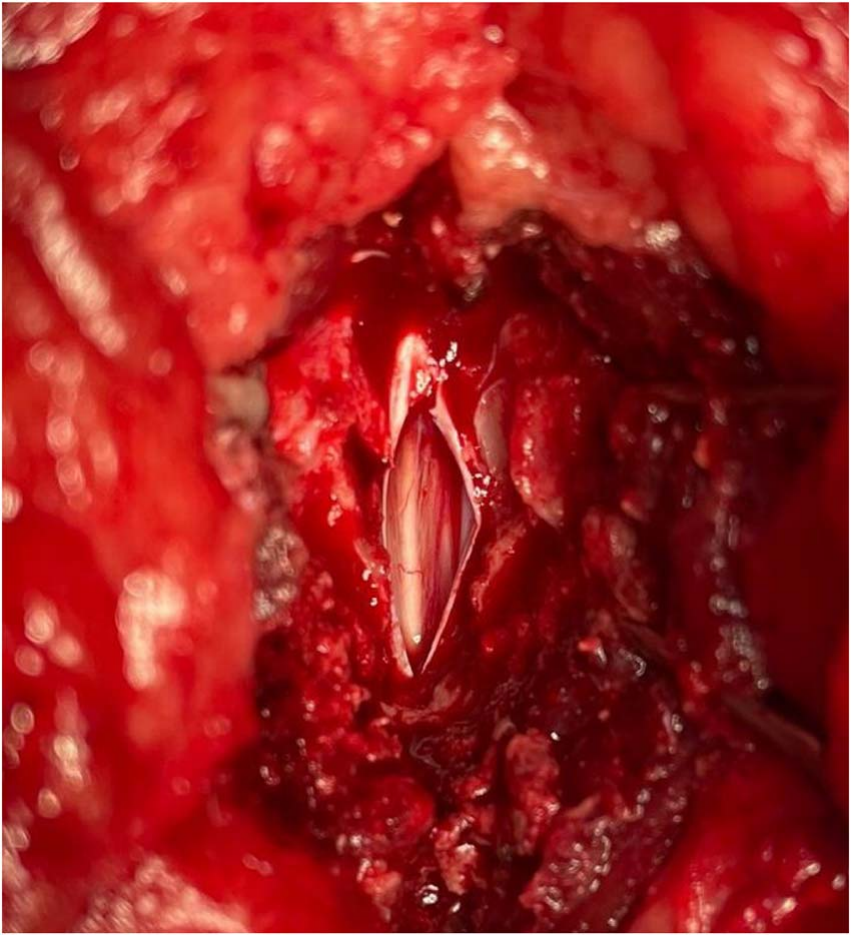
Intraoperative picture of the subarachnoid diverticulum, after durotomy. The filum terminale internum can be visualized.

Because of the chronic nature of the presentation and lack of response to analgesic medications, the owners elected for surgical intervention. The dog received premedication of methadone (0.2 mg/kg IV) and medetomidine (2 μg/kg IV), and general anesthesia was induced with propofol (1.6 mg/kg IV). Before surgery, an erector spinae plane block was performed using bupivacaine (2 mg/kg). General anesthesia was maintained with isoflurane and a continuous rate infusion (CRI) of ketamine (10 μg/kg/min IV). A dorsal laminectomy was performed at L6-7 using a high-speed burr and Kerrison rongeurs. Intraoperatively, a cystic structure was visualized displacing the epidural fat, which was no longer visible at this level. The structure was closely adhered to the cauda equina and upon inspection could not be safely excised (Figure 3). Macroscopically, the structure was consistent with a SAD (Type III). The lesion had a tail at its most caudal point and was closely associated with the left-sided nerve roots. A durotomy approximately 2 cm in length was performed over the lesion, resulting in the release of CSF and collapse of the diverticulum. Within the diverticulum, the filum terminale internum could be visualized (Figure 4)^10^ and the left-sided L6 nerve root was closely adhered ventrally. No additional leptomeningeal adhesions were discovered after gentle probing of the dural sac. Because of concern that marsupialization could place excess tension and potentially cause neurologic damage, this procedure was not performed. No meningeal closure was performed.

The dog was re-examined 3 h postoperatively, at which time no lameness was observed. However, the dog was receiving ketamine (5 μg/kg/min IV) and methadone (0.2 mg/kg IV q4h). The dog subsequently was tapered off injectable medications and discharged three days later with gabapentin (10 mg/kg PO q8h) and prednisolone (0.3 mg/kg PO q48h) for 7 days as part of a tapering regimen. During this period, the dog remained comfortable with no evidence of lameness.

A re-examination appointment was recommended for three weeks’ post discharge, but the owners did not return the dog. The owners were contacted eight weeks post-surgery, at which time they reported the dog was normal, with no evidence of pain or lameness. Video footage was assessed, and no evidence of lameness was seen.

## Discussion

We report a case of a SAD at the level of L6-7 in a 4-year-old Cockapoo. In a previous case, a French bulldog was presented with ambulatory paraparesis without signs of pain, which aligns with the more typical clinical presentation of SAD, in which only a small proportion of cases (18.9%) are presented for pain.^8^^,^^9^

The dog’s primary presenting sign was chronic hindlimb lameness and pain. The presentation resembled that described previously,^11^ in which 3 German shepherd dogs were presented with unilateral hindlimb lameness and lumbosacral pain associated with an extradural synovial cyst compressing the cauda equina and associated nerve roots.^11^ The SAD reported here was closely associated with the left-sided nerve roots, potentially explaining the pain and lateralization observed. However, the owners reported signs affecting both hindlimbs and we suspect that the SAD was compressing the cauda equina and resulting in a bilateral neurogenic lameness. In human medicine, Type III SAD are well documented as causes of radiculopathy when associated with nerve root compression, particularly in the caudal lumbar region.^12–14^ The dog of our report was male, which aligns with previous studies reporting a sex predisposition for males.^6^^,^^8^^,^^15^

Subarachnoid diverticula frequently are associated with intervertebral disc disease or vertebral malformations.^8^^,^^16^ In a previous study, 21.3% of dogs with SAD had concurrent neurologic disease at the same or an adjacent site. This feature was most common in the previously reported pug and French bulldog, with the pug’s SAD hypothesized to be linked to caudal articular process dysplasia.^17^ Additionally, 58% of pugs were found to have intervertebral disc disease at the same or at a site adjacent to the SAD.^14^ Interestingly, our dog had a transitional vertebra at L7. Transitional vertebrae have been shown to be associated with an increased frequency of cauda equina syndrome.^18^ There was no evidence of nerve root enlargement or contrast enhancement on MRI. Additionally, if the transitional vertebra had been the primary cause of the clinical signs because of compression of the exiting nerve root at that level it is unlikely such marked and immediate postoperative improvement would have occurred. A mild intervertebral disc protrusion (Hansen Type 2) also was identified at the level of L7-S1, which was not treated surgically and also was considered unlikely to have caused any of the clinical signs.

In the previously reported French bulldog with a SAD at this location, the dog improved but did not recover completely. It was hypothesized that chronic changes had resulted in permanent neurologic deficits, as evidenced by the French bulldog’s ongoing ambulatory paraparesis and ataxia.^9^ In contrast, our case appeared to recover completely, with no clinical signs evident upon short-term re-assessment. This difference may reflect breed variability in prognosis but could be associated with a shorter duration of spinal cord nerve root compression, or with differences in post-operative re-assessment between the two cases.

Unfortunately, no tissue was available for histopathologic evaluation. Findings on MRI and intra-operative observations however were consistent with a Type III SAD. Another differential diagnosis considered because of the lesion’s location was a persistent ventriculus terminalis, which to our knowledge has not been reported in dogs. However, this diagnosis was deemed unlikely because the structure did not appear continuous with the central canal and seemed to compress it externally. Additionally, the lesion was lateralized and eccentrically positioned rather than midline, making it less consistent with a persistent ventriculus terminalis as described in humans.^19^^,^^20^ The marked clinical improvement after decompression of the SAD supports the causality between the lesion and the clinical signs observed. Other potential causes cannot be entirely excluded but appear much less likely. The SAD was treated by simple linear durotomy without marsupialization. Previous studies have found no correlation between marsupialization and clinical outcome.^14^ In a larger study that evaluated surgical management of SAD, 82% of dogs improved postoperatively, despite only 3/26 cases undergoing marsupialization of the dura.^21^

Subarachnoid diverticula should be considered as a differential diagnosis in dogs presenting with cauda equina signs. Our case had a clinical presentation resembling that of an intervertebral disc extrusion at the same site. Therefore, clinicians should be aware that SAD can mimic intervertebral disc extrusions in this region.

## Off-label antimicrobial declaration

Authors declare no off-label use of antimicrobials.

## Institutional animal care and use committee or other approval declaration

Authors declare no institutional animal care and use committee was needed. The owner of the dog gave permission for the case to be used for teaching and research purposes, having been referred to the university’s teaching hospital, and signing the terms and conditions of the Queens Veterinary School Hospital. The owner also gave verbal consent for it to be used as a case report.

## Abbreviations

CSF: cerebrospinal fluid
MRI: magnetic resonance imaging
SAD: spinal subarachnoid diverticulum
